# Veterinary Columns in Secular Journals

**Published:** 1895-05

**Authors:** 


VETERINARY COLUMNS OF THE SECULAR JOURNALS.—There comes a time when the "worm will turn" and when the American citizen feels that there is a limit to his endurance. The morning papers of April 27th, which inform us that England has landed soldiers on the soil of a sister republic, to control the transit of the Atlantic to the Pacific, as she does that of the Mediterranean to the Indian Ocean, without protest from the Chief Executive of the United States, irritates one to be critical for the remainder of the day.

THE following article from the veterinary column of the *Turf, Field and Farm*, April 26, 1895, is sufficient to disgust the intelligent veterinarians of the country, who, while making their living by hard work, spend much of their time in trying to improve the status of the profession :

"UNUSUAL PHENOMENA.—I send by to-day's express a stone taken from Stella by Vauxhall. My son, who witnessed the post-mortem, says the stone was found in the immediate region of the lungs and heart, and rather between the two. My groom, who opened the mare, says he examined for the liver and could find none. Can it be that the liver had become petrified? The mare was apparently in good health and splendid condition for breeding. She ate all of her oats the night before and about half of her hay. On the next morning the groom opened her stable door about light, and she nickered to him as usual, but when he went to feed her he found she would not eat. She died about 10 A. M. R. J. H.

"*Answer*.—The character of this specimen presents somewhat the nature of a calcareous deposit, often found in the abdominal cavity in the form of hard, rounded bodies described as *calculi*, and composed of carbonates, phosphates, etc., deposited in concentric layers around some hard and indigestible substance that finds its way in the stomach and bowels.

"Finding this, however, in the pleural cavity, where such substances are seldom seen, in connection with the fact of there being no liver, makes this both a complicated and interesting case. The form of the specimen will readily admit of the theory that this is a metamorphosis of that organ into its present form. But we have to bear in mind that the liver has no business in the place where this seems to have been found. Although it is possible, it may, in its remarkably rough state, have so irritated the dividing wall as to cause a breakdown of that tissue, which would admit of its finding a location where it seems to have been found. Another remarkable feature of the case stares us in the face, that the mare must have lived a long time, certainly years, and apparently done well without a liver, a circumstance that even scientists will not admit can exist, as this remarkable change could not be brought about except through the medium of long time. We arrive at this conclusion from the fact there is no trace of any cespituous tissue about this foreign body, and absorption, always a slow process, would undoubtedly be the only means through which this would be accomplished."

If R. Y. H. wants to know about Stella why does he not spend a dollar or two, equal to what he puts in the church plate on Sundays, and employ a competent veterinarian to make an autopsy? What do his "son" and "groom" know of the anatomy of a horse, and what information of value does he expect to obtain from the veterinary editor of the *Turf, Field and Farm*, who finds from their description a mass in the "pleural cavity," and who accepts their statements as a fact that the horse in question had no liver? The veterinary expert(?) qualifies by "bearing in mind that the liver has no business in the place where it seems to have been found," "although it is possible," etc. [The liver to have been metamorphosed, to disappear from the right side of the abdominal cavity, pass through the diaphragm after severing its vascular attachments, pass obliquely to the left, through the mediastinum and be found "rather between" the lungs and heart.] Will the veterinarian(?) of the *Turf, Field and Farm* give us any authority for accepting that any mammal ever "lived a long time . . . without a liver," or that such an animal ever existed? It is rot. Let secular journals keep to the valuable matter which they should and do furnish, and which is of interest and value to their readers, and when their readers want to know about religion let them go to church and pay for their pew, and when they want to know about diseases of an animal let them go to a reputable veterinarian and pay him his fee.

